# Inducible promoters and functional genomic approaches for the genetic engineering of filamentous fungi

**DOI:** 10.1007/s00253-018-9115-1

**Published:** 2018-06-02

**Authors:** Janina Kluge, Dominik Terfehr, Ulrich Kück

**Affiliations:** Lehrstuhl für Allgemeine und Molekulare Botanik, Ruhr-University Bochum, Universitätsstr. 150, 44780 Bochum, Germany

**Keywords:** Fungal biotechnology, Inducible promoter systems, Heterologous products, Functional genomics, Strain improvement

## Abstract

In industry, filamentous fungi have a prominent position as producers of economically relevant primary or secondary metabolites. Particularly, the advent of genetic engineering of filamentous fungi has led to a growing number of molecular tools to adopt filamentous fungi for biotechnical applications. Here, we summarize recent developments in fungal biology, where fungal host systems were genetically manipulated for optimal industrial applications. Firstly, available inducible promoter systems depending on carbon sources are mentioned together with various adaptations of the Tet-Off and Tet-On systems for use in different industrial fungal host systems. Subsequently, we summarize representative examples, where diverse expression systems were used for the production of heterologous products, including proteins from mammalian systems. In addition, the progressing usage of genomics and functional genomics data for strain improvement strategies are addressed, for the identification of biosynthesis genes and their related metabolic pathways. Functional genomic data are further used to decipher genomic differences between wild-type and high-production strains, in order to optimize endogenous metabolic pathways that lead to the synthesis of pharmaceutically relevant end products. Lastly, we discuss how molecular data sets can be used to modify products for optimized applications.

## Introduction

Filamentous fungi possess a highly important role in pharmaceutical and biotechnical applications. As producers of primary and secondary metabolites, they are economically important for the food, paper, or pharmaceutical industry, among others (Alberti et al. 2017; Dufosse et al. 2014; Kuivanen et al. 2015). In order to obtain strains with an optimal production yield, strain improvement programs were set up to generate randomly mutagenized derivatives that have passed strict selection procedures. With the advent of DNA-mediated transformation systems for filamentous fungi about 40 years ago, in vitro recombinant technologies were invented to genetically manipulate industrial strains (Case et al. 1979; Stahl et al. 1982). While bacterial and yeast expression systems were accompanied by the development of sophisticated gene expression systems in the early studies, filamentous fungi lag behind in providing comparable systems (Alberti et al. 2017). This is in part due to the fact that the expression machinery is more complex in filamentous fungi than in bacteria and yeasts.

As an advantage, filamentous fungi tend to have post-translational modification systems that allow the production of functional mammalian proteins, which are difficult to perform in bacterial and yeast expression systems in many cases (Ward 2012).

In a recent study, molecular tools, such as homologous recombination and RNA interference systems that are suitable for the genetic manipulation of filamentous fungi were reviewed (Kück and Hoff 2010). Here, we will extend this survey by summarizing the available inducible promoter systems to obtain controlled gene expression in industrial strains. Finally, we focus on representative examples, where filamentous fungi were used for the successful production of heterologous proteins. Nowadays, this is highly influenced by the availability of genomic and functional genomic data sets, which are used for novel strategies in strain improvement programs. Another recent valuable tool for directed strain improvement is the CRISPR/Cas9 system, which is suitable for multiple gene editing events. We would like to refer to recently published reviews that cover the development of CRISPR/Cas9-based gene editing in filamentous fungi (Krappmann 2017; Shi et al. 2017).

### Claims and conditions for the application of inducible promoters

Extensive studies in eukaryotic systems have established the mechanistic key concept of controlled gene regulation (Levine et al. 2014; Soutourina 2017; Spitz and Furlong 2012). The expression of eukaryotic genes is governed primarily at the level of transcriptional initiation, which corresponds to the complex interplay between the promoter, RNA polymerase II, and transcription factors (Sainsbury et al. 2015; Struhl 1987). Promoters are defined as the DNA sequence immediately surrounding the transcription start site, which is bound by the basal transcription machinery, thus allowing transcriptional initiation. Over the years, the knowledge of promoter architecture expanded and led to the development and establishment of constitutive and inducible promoter systems (Su et al. 2012; Woo and Li 2011). In Table 1, we list inducible promoter systems that are commonly used in applied research with filamentous fungi. In the following, some of these are described in detail.

**Table 1:** Inducible promoters in filamentous fungi

Promoter	Gene product	Induction by/repression by	Donor	Reference
*alcA*	Alcohol dehydrogenase I	Ethanol/glucose	*Aspergillus nidulans*	Waring et al. (1989)
*amyB*	TAKA-amylase A	Starch or maltose/glucose	*Aspergillus oryzae*	Tada et al. (1991), Tsuchiya et al. (1992)
*bli-3*	Blue light-inducible gene	Blue light/darkness	*Neurospora crassa*	Eberle and Russo (1994)
*bphA*	Benzoate p-hydrolase	Benzoic acid (benzoate)/deficiency of benzoate	*Aspergillus niger*	Antunes et al. (2016)
*catR*	Catalase	H_2_O_2_ and CaCO_3_/n.d.	*Aspergillus niger*	Sharma et al. (2012)
*cbhI*	Cellobiohydrolase I	Various saccharides/glucose	*Trichoderma reesei*	Nyyssönen and Keränen (1995)
*cre1*	Glucose repressor	Glucose/deficiency of glucose	*Acremonium chrysogenum*	Janus et al. (2008)
*exylA*	Endoxylanase	Xylose/sucrose	*Aspergillus awamori*	Gouka et al. (1996)
*gas*	1,3-beta-glucanosyltransferase	Low pH value/pH > 5.0	*Aspergillus niger*	Yin et al. (2017)
*glaA*	Glucoamylase A	Glucose/xylose	*Aspergillus niger*	Boel et al. (1984)
*gla1*	Glucoamylase	Glucose/xylose	*Penicillium verruculosum*	Bulakhov et al. (2017)
*mir1*	Siderophore transporter	Iron starvation/Iron sufficiency	*Acremonium chrysogenum*	Gsaller et al. (2013)
*niiA*	Nitrite reductase	Nitrate/ammonium	*Aspergillus oryzae*	Müller et al. (2002)
*qa-2*	Catabolic 3-dehydroquinase	Quinic acid/low quinic acid or high sugar concentration	*Neurospora crassa*	Giles et al. (1985)
*Smxyl*	Endoxylanase	Xylose/glucose	*Acremonium chrysogenum*	Bloemendal et al. (2014)
*tcu-1*	Copper transporter	Copper depletion/copper availability	*Neurospora crassa*	Lamb et al. (2013)
*thiA*	Thiamine thiazole synthase	Thiamine/deficiency of thiamine	*Aspergillus oryzae*	Shoji et al. (2005)
*vvd*	Blue light receptor	Light/darkness	*Neurospora crassa*	Hurley et al. (2012)
*xyl1*	Endoxylanase	Xylose (and xylan)/glucose	*Acremonium chrysogenum*	Blatzer et al. (2014)
*xylP*	Endoxylanase	Xylose (and xylan)/glucose	*Penicillium chrysogenum*	Zadra et al. (2000)
*xyn1*	Endoxylanase	Low concentration of xylose/high concentration of xylose	*Trichoderma reesei*	Mach et al. (1996)
*zeaR*	Transcription factor	Zearalenone/Deficiency of zearalenone	*Fusarium grainearum*	Lee et al. (2010)

Inducible promoters were initially developed to synthesize exactly one additional gene product under precisely defined conditions within a certain time interval. Therefore, these systems find application in the functional characterization of essential genes in the laboratory, as well as in the biotechnological production of heterologous protein products, where a fine-tunable expression is often desired, especially when the protein is toxic to the cell (Lee et al. 2010; Nevalainen et al. 2005). An ideal inducible promoter is primarily characterized by a strong and tight controllable regulation, a cost-efficient induction, and an effective expression of the gene of interest being placed downstream of the inducible promoter sequence. In general, a distinction takes place between chemically and physiologically induced promoters. Chemically regulated systems are induced or repressed by the presence or absence of chemical compounds, such as alcohols, antibiotics, hormones, or carbon sources (Matsuzawa et al. 2013; Meyer et al. 2011). In contrast, the regulation of physiologically controlled promoters is determined by abiotic environmental factors. In this context, systems were established, whose regulation takes place depending on the osmotic stress, temperature, light, etc. (Fischer et al. 2016; Zhang et al. 2016).

The selection of a suitable promoter system should be specifically adapted to experimental approaches because not every system brings along optimal conditions for the selected model organism. Thus, it must be considered that certain inductive or repressive substances or the produced protein has a toxic effect in some organisms (Kim et al. 2016; Lee et al. 2010). In addition, a leakiness of the promoter, which results in a minimal constitutive activity, leads to uncontrolled gene expression (Meyer et al. 2011). In the subsequent years, several inducible expression systems were discovered and used for applied approaches. Few of them that were used in recent biotechnical applications will be summed up in this review.

### Inducible promoters depending on carbon sources

The *glaA* promoter of the glucoamylase A gene from *Aspergillus niger* was one of the first inducible systems, which was commonly used in filamentous fungi. Induction of *glaA* promoter-driven gene expression occurs on starch-containing growth media or alternatively on media with maltodextrine, maltose, or glucose. Repression is achieved when strains are grown on xylose-containing minimal media (Siedenberg et al. 1999). In another homologous promoter system, promoter sequences of the glucoamylase gene were fused with the β-glucosidase gene from *A. niger* or endoglucanase IV from *Trichoderma reesei* for the construction of recombinant *Penicillium verruculosum* strains (Bulakhov et al. 2017). Compared to the wild type, the strain harboring the recombinant promoter displayed a more efficient hydrolysis of a lignocellulosic substrate. In *Aspergillus oryzae*, the *glaA* promoter was recently used for the production of L-malate, which is widely utilized in the food and beverage industry as an acidulant and flavor enhancer (Liu et al. 2017b; Thakker et al. 2015). A further improvement of promoter strength was attained when five copies of the -427 to -331 upstream region of *glaA* carrying at least one CAAT-Box were integrated to efficiently increase L-malate production directly from corn starch (Liu et al. 2017b). This cis-element arrangement was also shown to improve promoter strength and inducibility of the *Trichoderma reesei cbh1*and *xyn1* promoters. Detailed analysis of the first 850 bp of both promoter sequences identified binding sites for Xyr1 (XBS), the essential transactivator of *cbh1* and *xyn1* gene expression. In these promoters, 14 and 8 XBS, respectively, are arranged in tandem or in the inverted repeat direction. Further improvement of these promoters was obtained when additional XBS were integrated in inverted repeat orientation. In the case of *cbh1* promoter, an induction of gene expression by xylan or wheat straw was received by modifying the XBS inverted repeats (Kiesenhofer et al. 2018). Also, in a recent attempt, the *cbhI* promoter was further improved to control the heterologous expression of two feruloyl esterase genes to produce ferulic acid, a food additive or potential anti-inflammatory therapeutic agent from wheat bran (Long et al. 2018).

Xylose-inducible promoters have been established in some industrial host systems for some time, including the abovementioned *xyn1* promoter from *T. reesei* and *xylP* from *Penicillium chrysogenum* (Zadra et al. 2000). Interestingly, the latter was proven to be functional in other filamentous fungi from the order Sordariomycetes (Bloemendal et al. 2014; Kopke et al. 2010). This includes the well-established β-lactam producer *Acremonium chrysogenum* for which three controllable promoter systems were developed in recent years. One is the *mir1* promoter, encompassing a 1700-bp region upstream of a siderophore transporter gene, which was shown to induce *gfp* gene expression during iron starvation, while iron sufficiency results in repression (Gsaller et al. 2013). Two other inducible promoters stem from the xylanase genes from *Sordaria macrospora* and *A. chrysogenum* and drive gene expression on xylose-containing media. On the contrary, glucose results in gene repression. Both promoters are very effective in *A. chrysogenum* and contribute to the rather limited number of molecular tools for this highly important industrial fungus. Furthermore, the *Smxyl* promoter was successfully used for the application of a one-step FLP/*FRT* recombination system in order to construct marker-free transgenic strains (Blatzer et al. 2014; Bloemendal et al. 2014).

### Metabolism-independent inducible promoter systems

A few years ago, the thiamine-regulatable *thiA* promoter was established for *A. oryzae*. The expression level of this promoter was controlled by the concentration of external thiamine in the media. This promoter was recently used in an interesting approach, where autophagy-related genes were shown to affect the heterologous expression of the bovine chymosin gene in *A. oryzae*. The transformation of *A. oryzae* strains, lacking autophagy genes *Aoatg1*, *Aoatg13*, *Aoatg4*, *Aoatg8*, or *Aoatg15* with a bovine chymosin (CHY) expression construct resulted in a threefold enhanced production level of CHY in comparison to the control strain. However, conidiation was significantly reduced in these strains. Since huge amounts of conidia are necessary for the inoculation of large-scale cultures, gene-conditional expression strains of four *Aoatg* genes were constructed in which the promoter region of the corresponding genes was substituted by the *thiA* promoter. Conidiation was clearly increased in the absence of thiamine, whereas autophagy was still repressed under growth conditions with thiamine. Nevertheless, the production level of CHY was comparable to that of the ∆Aoatg strains (Yoon et al. 2013). The fact that thiamine content does not alter fungal morphology makes the *thiA* promoter a useful alternative to conventionally used promoters. For example, *alcA* and *amyB* promoters depend on the available carbon sources, which might have an impact on fungal physiology or morphology (Shoji et al. 2005). In submerged cultures from *A. nidulans*, expression of the *abaA* gene under the control of the *alcA* promoter resulted in vacuolated hyphae with abnormal septation (Mirabito et al. 1989). A comparable effect was observed when a copper-inducible promoter was used for heterologous expression since copper has physiological and toxic effects on fungal growth and development (Lee et al. 2010).

A completely new promoter system was established for *A. niger.* This was adapted by the medium conditions of citric acid fermentation. During this process, the pH of the growth medium decreased from 5.0 below 2.0 and was nearly consistent at a value below 2.0 in later cultivation phases. Therefore, a pH-responsive promoter, P*gas*, which efficiently enhances gene expression at pH 2.0, was developed for dynamic metabolic engineering. The *gas* gene was found in an expression data-set and encodes the 1,3-β-glucanosyltransferase GelD, which is necessary for maintenance of the fungal cell wall due to the synthesis of glucan. This study showed that P*gas* is tightly expressed at pH 2.0. In terms of promoter strength, P*gas* is comparable to P*gpdA*. Furthermore, the promoter strength varied by using different types of acids. While induction was strictly pH dependent, promoter strength was higher using organic acids compared to inorganic acids. P*gas* was used to express the cis-aconitate decarboxylase (*CAD*) gene from *Aspergillus terreus* in *A. niger*. Due to the fact that *A. niger* produces large amounts of citrate (180 g/l), which is the precursor of itaconate, the aim was to modify the natural citrate producer into an itaconate producer. Itaconic acid is an unsaturated dicarbonic acid with a high industrial potential because it can be used as a monomer for the production of a plethora of products including resins, plastics, paints, and synthetic fibers. Since constitutive expression of the *CAD* gene resulted in only a small yield of itaconate, the use of the P*gas* promoter led to a gradually increased expression level of *CAD* parallel to a decreasing pH value*.* Finally, a titer of 5 g/l was obtained, which was fivefold higher than comparable experiments in which the P*gpdA* promoter was used to drive expression of the *CAD* gene (Yin et al. 2017).

### Adaption of the Tet system for eukaryotes

**Fig. 1:**
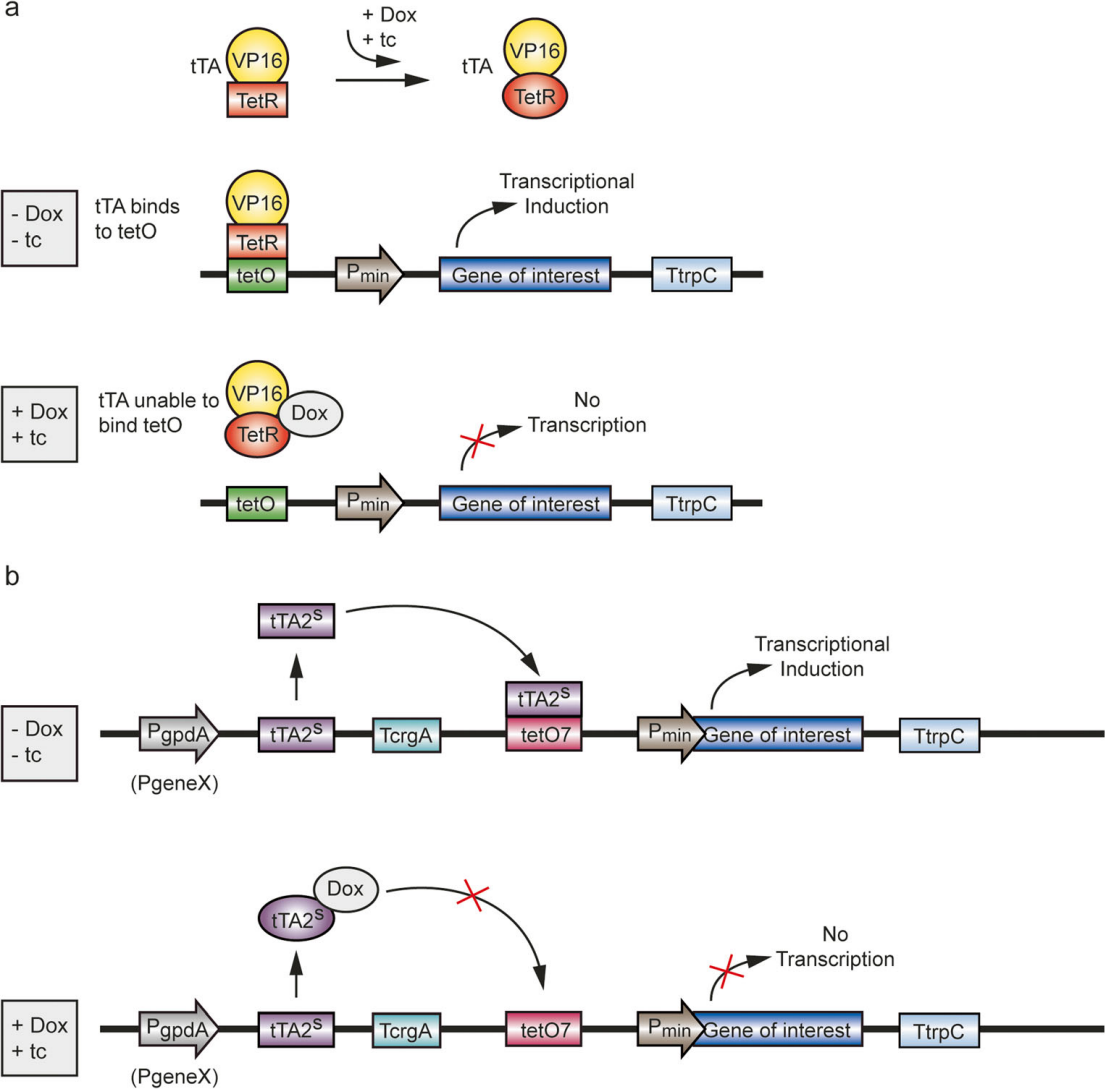
Tet-Off expression system. **a** Basic version of the Tet-Off expression system in eukaryotes. The fusion of TetR with VP16 leads to the generation of the tetracyclin-dependent transactivator tTA. When tetracycline or its analog doxycycline is added, tTA is unable to specifically bind to tetO due to a conformational change. As a result, transcription of the gene of interest, which is controlled by P*min*, is disrupted. Thus, transcriptional activation takes place in the absence of tetracycline or doxycycline. Addition of the either drug leads to transcriptional shutdown. Dox, doxycycline; VP16, transcriptional activator domain from herpes simplex virus; tc, tetracycline; TetR, tetracycline-dependent Tet repressor; tetO; tetracycline operator sequence; tTA, tetracyclin-dependent transactivator; T*trpC*, *trpC* terminator from *Aspergillus nidulans*; Pmin, minimal promoter. **b** Optimized module of the Tet-Off expression system for *A. niger*. The transactivator tTA2^S^, optimized for mammals, was used downstream of P*gpdA* promoter. The promoter can be selected individually depending on the model organism (P*geneX*). Seven copies of tetO sequence were inserted (tetO7) upstream of a minimal promoter in order to increase tTA2^S^ binding. In the absence of tetracycline or its analog doxycycline tTA2^S^ is able to specifically bind tetO7. Thus, the transcriptional induction of the gene of interest, which is controlled by P*min* takes place. Addition of drug leads to transcriptional shutdown. For further details, see text. Abbreviations: Dox, doxycycline; VP16, transcriptional activator domain from herpes simplex virus tc, tetracycline; TetR, tetracycline-dependent Tet repressor; tetO, tetracycline operator sequence; tetO7, seven copies of tetracycline operator sequence; tTA, tetracyclin-dependent transactivator; tTA2^S^, mammalian tetracyclin-dependent transactivator; T*crgA*, *crgA* terminator from *Aspergillus fumigatus*; T*trpC*, *trpC* terminator from *Aspergillus nidulans*; P*min*, minimal promoter (adopted from Brockamp et al. 2002, Wanka et al. 2016)

**Fig. 2:**
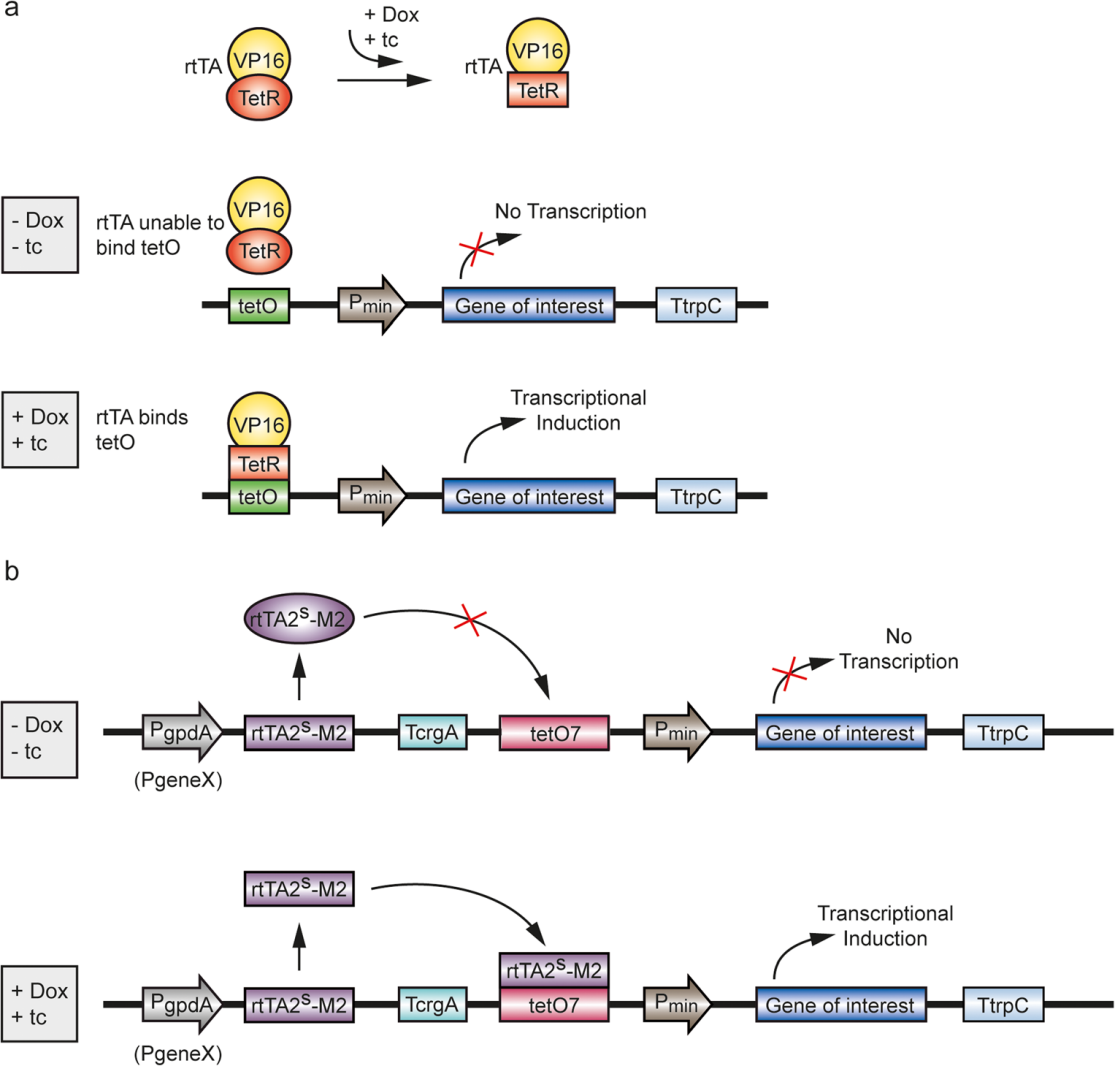
Tet-On expression system. **a** Basic version of the Tet-On expression system in eukaryotes. The rtTA fusion protein comprises of TetR and VP16 activation domain. A four amino acid change in the TetR DNA binding motif alters rtTA’s binding characteristics, such that it can only recognize tetO sequence in the presence of tetracycline or its analog doxycycline. Thus transcriptional induction of the gene of interest, which is controlled by a minimal promoter, takes place. **b** Optimized version of the Tet-On expression system for *A. niger*. The insertion of additional mutations resulted in the reverse hybrid transactivator rtTA2^S^-M2, which is controlled by the P*gpdA* promoter, to increase the rtTA binding sensitivity and the expression level of rtTA. The used promoter can be selected individually depending on the model organism (P*geneX*). An improvement of tetO was achieved by the insertion of seven copies of tetO sequence (tetO7) upstream of the minimal promoter. In the presence of tetracycline or its analog doxycycline, rtTA2^S^-M2 is able to specifically bind tetO7 in order to induce the transcription of the selected gene of interest. For further details, see text. Abbreviations: rtTA2^S^-M2, mutated reverse hybrid transactivator. All other abbreviations are given in the legend of Fig. 1. (adopted from Brockamp et al. 2002, Wanka et al. 2016)

Another frequently used expression mechanism, which was originally identified in *Escherichia coli* is the tetracycline (tc) expression system (Tet system). The central components of this system stem from the Tn10 tetracycline resistance operon. In Gram-negative bacteria, the membrane-residing TetA protein is responsible for an antiport of tc, which leads to resistance to this antibiotic. The *tetA* gene is under the transcriptional control of the tc-dependent Tet repressor (TetR), which negatively controls tc resistance. In the absence of tc, TetR binds to the tet operator (tetO) resulting in a transcriptional shutdown of the operon. In contrast, in the presence of tc, the interaction between the repressor and the operator is efficiently prevented, leading to a dissociation of TetR from tetO. For use in eukaryotic organisms, the modification of regulatory elements was necessary and a differentiation between a Tet-On and the Tet-Off system was made. Transgenic mice were the first eukaryotes in which Tet-Off was established (Gossen and Bujard 1992). A tc-dependent transactivator (tTA) was generated by the fusion of TetR with the transcriptional activator domain from herpes simplex virus protein 16 (VP16). By the addition of tc or its analog doxycycline (Dox), tTA cannot specifically bind to tetO; thus, transcription of the gene of interest is disrupted (Tet-Off state) (Fig. 1a). In contrast, for the Tet-On system, a reverse hybrid tTA (rtTA) was constructed, which, in the presence of tc or Dox, functions as an activator of gene expression. The insertion of additional mutations resulted in the reverse hybrid transactivator rtTA2^S^-M2 to increase the rtTA binding sensitivity to Dox and the expression level of rtTA in eukaryotes. An improvement of the tetracycline-responsive promoter was achieved by the insertion of seven copies of the tetO sequence (tetO7) upstream of a minimal promoter P*min* (Fig. 2a, b).

### Previous and recent applications of the Tet system

Previously, the Tet-On/Tet-Off system was shown to be functional in *Aspergillus fumigatus* (Vogt et al. 2005). In this study, three plasmids were constructed, one harboring the expression cassette consisting of the transactivator rtTA2^S^-M2 under the control of the constitutive *gpdA* promoter (P*gpdA*::rtTA2^s^-M2), the other comprising P*gpdA*::tTA, and the third one with a tetO7 sequence linked to a minimal promoter sequence of P*gpdA* upstream of an *E. coli* hygromycin resistance gene (tetO7::P*min*::hyg^R^). The corresponding plasmid pairs were transferred in parallel into a single *A. fumigatus* strain and hygromycin-resistant transformants were obtained in the presence and absence of Dox indicating that the Tet system functions in *A. fumigatus*. However, the tightness of the system was not examined in detail. Especially the Tet-On system was further developed for an application in *A. niger*. It was also established as a metabolism-independent gene expression system in this study, but all components of the Tet-On system were combined on one plasmid. For testing its functionality, a minimal *gpdA* promoter, which was fused to the tetO7 sequence, was used to control luciferase gene expression. While in the absence of Dox no luminescence was detectable, the presence of Dox led to the induction of luciferase gene expression. It was clearly shown that the system is tight, induction takes place immediately after addition of the inducer, and fine-tuning of the system is achieved by inducer concentration and gene copy number (Meyer et al. 2011).

In recent years, the Tet system was also used for the controlled expression of secondary metabolite clusters, such as silent gene clusters in *Aspergilli*. In *A. fumigatus* strains grown under induced conditions, the Tet-On overexpression of a putative transcription factor resulted in the activation of a secondary metabolite cluster, which led to the biosynthesis of 5-benzyl-1H-pyrrole-2-carboxylic acid, an unknown novel natural compound, named fumipyrrole (Macheleidt et al. 2015). In *A. niger*, the Tet-On system was used successfully for the expression of polycistronic genes from the Asp-melanin biosynthetic pathway from *Aspergillus terreus* (Geib and Brock 2017). This is a further good example that the expression genes from secondary metabolite clusters can be tightly expressed in heterologous hosts. Another remarkable example was reported recently for the rice pathogen *Fusarium fujikuroi.* In this fungus, constitutive overexpression of the transcription factor TF22 drives the activation of the complete trichosetin gene cluster. Trichosetin is a PKS-NRPS-derived tetramic acid possessing biological activity against a broad range of organisms, encompassing plants and bacteria. Constitutive overexpression resulted in severe growth reduction. To circumvent this undesired morphological defect, the Tet-On system from *A. niger* was established for *F. fujikuroi* to obtain the controlled production of trichosetin for further the functional characterization of each gene within the trichosetin cluster (Janevska et al. 2017). One part of the Tet construct contains the already described transactivator rtTA2^S^-M2 under the control of the constitutive P*oliC* promoter from *A. nidulans*, while the other part encompasses the rtTA2^S^-M2-dependent promoter in front of TF22. The pTET::TF22 plasmid was integrated into the wild type and five mutants, carrying deletions of different cluster genes. All transformants showed a strong activity of TF22 expression dependent on the added Dox concentration. However, a leakiness of the system was observed because *TF22* showed a basal expression without adding the inducer. As this background expression did not drive activation of the gene cluster, the Tet-On system was suitable as a controllable induction tool. The inducible overexpression of TF22 results in activation of the three cluster genes indicating that this transcription factor directs the biosynthesis of trichosetin.

Optimization of the Tet-Off system in *A. niger* was achieved by using the present plasmid for the Tet-On system and the reverse transactivator rtTA2^S^-M2 against the tTAS^2^ transactivator was exchanged, which is optimized for mammals, in order to construct a functional Tet-Off cassette (Fig. 1b) (Wanka et al. 2016). Strains with a single copy of the modulated Tet-Off showed a strong luciferase activity, whereas the addition of different Dox concentrations resulted in a downregulation of the luciferase gene expression. However, during the establishment of Tet-Off, it was apparent that not all *A. niger* transformants containing the Tet-Off construct show a luciferase downregulation after induction. Additionally, most of the transformants lost the Tet-Off cassette during storage on minimal medium. Genetic analysis of the transformants detected 176 bp sequence homology between the minimal *gpdA* promoter and the constitutive *gpdA* promoter and this resulted in intra-molecular recombinations and loss of function. Thus, constitutive P*gpdA* was replaced by the endogenous promoter of the *fraA* gene, encoding a putative ribosomal subunit. Using the P*fraA* Tet-Off system, genetically stable transformants were generated, and, in the presence of Dox, a rapid reduction in reporter luminescence was observed. In order to demonstrate a putative application for the characterization of industrially relevant genes, the luciferase reporter gene was substituted by the *gfaA* gene, which encodes a glutamine:fructose-6-phosphate aminotransferase. This enzyme is involved in the first step of chitin synthesis and chitin synthesis-defective strains cannot grow on media without the supplementation of glucosamine. The functionality of P*fraA* Tet-Off system-*gfaA* fusion was finally demonstrated when a *gfaA*-deficient strain was transformed with this construct. In the presence and absence of both Dox and glucosamine in the media, transformants are viable. However, the sole induction of the P*fraA* Tet-Off system by Dox, in the absence of glucosamine, results in the lethality of transformants. Thus, this system seems to be broadly suitable for industrial application (Wanka et al. 2016). Very recently, the Tet expression system was used to characterize genes that are involved in the viability of *A. fumigatus* during the infection process in order to identify new antifungal drug targets. For an in vivo approach, a Tet-Off system was designed where the presence of Dox resulted in the downregulation of gene expression. As potential target genes, those for inosine 5′-monophosphate dehydrogenase (*IMPDH*), which catalyzed the first step in de novo guanine biosynthesis, and L-ornithine N^5^-oxygenase (*sidA*), catalyzing the initial step of siderophore biosynthesis, were tested in a murine invasive pulmonary aspergillosis model. The immunosuppression of mice was induced by injection of cyclophosphamide for 4 days and 1 day before inoculation and injection of cortisone acetate 1 day before inoculation. On inoculation day, 40 μl of a fresh *A. fumigatus* conidia suspension was intranasally inoculated under anesthesia. The addition of 200 μl Dox (10 mg/ml) was carried out twice a day by gastric lavage. The downregulation of *impdh* resulted in reduced virulence in vivo, while switching off the gene expression of *sidA* led to the generation of avirulent *A. fumigatus* strains. Additionally, a time-dependent control of *sidA* expression was verified and the timing of Dox supplementation dramatically impaired the survival rate of mice. This study provides evidence that the Tet-Off system is used to identify genes required for fungal growth within the host following the initiation of infection (Peng et al. 2018).

## Production of heterologous products

Filamentous fungi are often used to produce endogenous compounds which are generated during the primary or secondary metabolism. Often, these enzymes are active within distinct metabolic pathways. Primary metabolites such as organic acids are used in diverse industrial applications. For example, citric, gluconic, fumaric, koji, lactic, and itaconic acids are valuable byproducts that are used in the chemical or food industry (Becker et al. 2015). Citric acid is within these fungal organic acids, the one with the highest yearly production yield (1.75 million tons), and has a market value of US$1.6 billion (Demain 2007). Besides organic acids, vitamins are important metabolites, which are produced with the help of filamentous fungi, e.g., *Ashbya gossypii* produces riboflavin (vitamin B2) at a yield of 20 g/l. In addition to primary metabolites, filamentous fungi are potent producers of enzymes of which about 100 are used for industrial applications. Most of these enzymes show a hydrolytic activity, such as amylases, proteases, lipases, xylanases, or catalases and are mostly used for the degradation of organic material. Amylases have the highest importance in this context, with an annual production of 95,000 tons and a market value of US$2.5 billion (Demain and Vaishnav 2009). Many of these enzymes are heterologously produced in diverse microbial host systems to circumvent difficulties in the cultivation and genetic manipulation of exotic microbes (Schmidt-Dannert 2015). Many fungal enzymes are synthesized in heterologous fungal host that are already established as industrial producers for a wide range of different enzymes or primary- and secondary metabolites. A list of commercial enzymes produced using filamentous fungi can be found at the Association of Manufacturers and Formulators of Enzyme Products (AMFEP, www.amfep.org). Fungi usually have a high secretion capacity and many production strains have a GRAS (generally recognized as safe) status. Furthermore, the post-translational modification of proteins is distinct in eukaryotic and prokaryotic host systems. Established fungal host systems that are amendable for genetic manipulation and with a GRAS status are *A. niger*, *A. oryzae*, and *T. reesei* (Schmidt 2006; Sharma et al. 2009). Therefore, filamentous fungi were used in several efforts for the production of mammalian enzymes and proteins. Particularly, the post-translational modification in eukaryotic microbes has supported attempts to obtain functional proteins from fungal hosts. The first successful approaches with filamentous fungi demonstrated the production of chymosin and lactoferrin (Archer 2000). Chymosin from calf vells is used in the food industry for the production of cheese, while lactoferrin, a multifunctional transferrin from cow’s milk, has an anti-inflammatory activity. Meanwhile, yeast expression systems have been shown to be superior for the biotechnical production of mammalian proteins with pharmaceutical relevance. Most common host organisms are *Saccharomyces cerevisiae*, *Pichia pastoris*, and *Hansenula polymorpha* (Ganeva et al. 2017; Mallu et al. 2016; Shibui et al. 2013). Nevertheless, filamentous fungi are still used for the heterologous production of different mammalian proteins, for example, the human hormone peptide obestatin was successfully produced using recombinant *T. reesei* strains (Sun et al. 2016). Recently, two fusion proteins each consisting of a highly antigenic protein from *Leishmania sp.*, an antibody specific for a receptor of dendritic cells, and a fragment of native glucoamylase A were generated in *A. niger* (Magana-Ortiz et al. 2018). Both recombinant proteins may be used for immunization against Leishmaniasis and point to the potential of filamentous fungi as hosts for the generation of pharmaceutically relevant peptides. In this context, it has to be mentioned that well-established organisms with beneficial traits are redeveloped for recombinant protein production. *Neurospora crassa* is a model organism with suitable traits for heterologous production but has not widely been used for industrial applications. In order to evaluate the usability of *N. crassa* as a host system, the biosynthesis of the human antibody fragment HT186-D11 was tested and production yields of about 3 mg/L were achieved (Havlik et al. 2017). Although the achieved yields cannot compete with established production hosts like *P. pastoris*, which is able to produce up to 100-fold higher yields, *N. crassa* seems to be a promising new host system for the production and secretion of heterologous proteins.

## Strain improvement using genomics and functional genomics

The strain improvement programs of *A. chrysogenum*, *P. chrysogenum,* and *T. reesei* are suitable examples for the efficiency of classical strain improvement by random mutagenesis (Hu and Zhu 2016; Peterson and Nevalainen 2012). However, the random introduction of point mutations also has disadvantages. Improved production strains can acquire point mutations over several rounds of random mutagenesis leading to a reduced growth rate, sporulation defects or genomic instability (Künkel et al. 1992; Lein 1986). In this context, directed genome manipulation can help to overcome the effect of deleterious point mutations. However, an essential prerequisite for this approach is the knowledge of the genome sequence, the encoded genes and the derived metabolic pathways. Therefore, functional genome analysis is an important tool with which to obtain the required information. Within functional genome analysis, the large data amounts of data from different high-throughput techniques are used to assign gene functions with the overall goal of analyzing the relation between the genotype and phenotype of an organism on a genome-wide level.

The still-progressing development of the next-generation sequencing techniques allows a fast and low-cost effective sequencing of complete fungal genomes for which the costs were reduced by a factor of about 50,000 (Goodwin et al. 2016). In parallel, efficient sequencing techniques have increased the output of sequences per run by a factor of 100 to 1000. The newest sequencing platforms, such as Pacific Biosciences (PacBio) or Oxford Nanopore Technologies (ONT), provide continuous sequences with a size of up to 200 kb. This is of major importance when repetitive regions carrying telomers or centromeres have to be sequenced. Currently, about 2700 genomes of fungal species are published and are represented, for example, in the 1000 Fungal Genome Project (http://1000.fungalgenomes.org/home/). The sequenced genomes possess a large number of uncharacterized genes, whose functions can be sometimes deduced from orthologous sequences known from other species. Valuable classification libraries to evaluate the function of proteins include the Functional Catalogue (FunCat), Gene Ontology (GO), or the Kyoto Encyclopedia of Genes and Genomes (KEGG) (Rüpp et al. 2004, Ashburner et al. 2000, Karnehieser and Goto 2000). A common characteristic of these databases is that classifications of genes are done on the basis of hierarchical structures, in which major categories are split up into sub-categories. Each sub-category provides more precise information, e.g., about the cellular function of the protein. An alternative approach in predicting the function of an unknown protein is the use of conserved protein domains for a homology-based search. These domains can be used to search against databases like Protein families (Pfam), Clusters of Orthologous Groups of proteins (COGs), and The Institute for Genomic Research’s database of protein families (TIGRFAM) to identify other proteins with similar domains or domain structure and to predict protein functions (Finn et al. 2016; Galperin et al. 2015; Haft et al. 2003). With this kind of homology-based functional analysis, even genomes of uncharacterized organisms, metagenomes, and metatranscriptomes can be analyzed. Representative examples from the field of filamentous fungi are the genomes from *Lichtheimia corymbifera*, a member of the basal Mucorales, or various *Penicillium* species (Lessard et al. 2014; Nielsen et al. 2017; Schwartze et al. 2014). The genome sequences from 24 *Penicillium* species revealed that a total of 1317 putative biosynthetic gene clusters exist. This survey indicates the potential of natural products that can be synthesized by members of this genus (Nielsen et al. 2017). After identification, interesting genes or even whole gene clusters can be expressed heterologously in suitable host systems (Bok et al. 2015).

Similar to metagenomes, which are generated by using total DNA from the sample for high throughput sequencing, metatranscriptomes are generated from the RNA of a given sample. The obtained sequencing data will identify active metabolic pathways that are effective in biotechnical processes. For example, the metatranscriptome of *Penicillium camemberti* and *Geotrichum candidum*, which are used for the production of Camembert-type cheese, was taken over 77 days of ripening. The functional annotation identified processes during Camembert ripening, such as carbohydrate or protein metabolic processes, which are most active in the first 2 weeks of ripening. Based on these data, attempts can be undertaken to improve cheese quality assessment (Lessard et al. 2014).

### Identification of biosynthesis genes and their related metabolic pathways

An essential aspect for biotechnological applications is knowledge of the genes involved in the biosynthesis of a given product, as well as detection of the metabolic pathways that provide the required precursors. However, when the functional assignment of genes and the related pathways are unknown, it can be achieved by functional genomics approaches. Transcriptomic data of *A. oryzae* strains inoculated in media with different kojic acid biosynthesis rates were used for comparative analysis, in order to identify genes involved in the biosynthesis of this important organic acid (Terabayashi et al. 2010). Identification of the involved genes allows targeted strain improvement by the overexpression of the related biosynthesis genes. Most biosynthesis pathways contain one or more rate-limiting steps representing interesting adjustment screws for product titers. For example, the biosynthesis of the phytohormone gibberellic acid produced by *F. fujikuroi* was enhanced by the overexpression of two genes encoding a geranylgeranyl diphosphate synthase 2 and a bifunctional ent-copalyldiphosphate synthase/ent-kaurene synthase. Both are key enzymes of the gibberellic acid pathway (Albermann et al. 2013). For *P. chrysogenum*, it was shown that even in production strains, the additional overexpression of *penDE* can lead to elevated penicillin yields (Weber et al. 2012). The acyl-coenzyme A: isopenicillin *N* acyltransferase encoded by *penDE* is involved in the last biosynthetic step of penicillin formation, which is the exchange of the sidechain L-α-aminoadipic acid with phenylacetic acid or phenoxyacetic acid. However, the overexpression of *penDE* needs to be balanced since very high levels of the encoded enzyme lead to accumulation of 6-aminopenicillic acid instead of penicillin. In this case, the formation of 6-aminopenicillic acid by uncontrolled removal of the sidechain of penicillin occurs probably due to an insufficient supply with sidechain precursors. The supply of precursors is also a very promising field for strain improvement. The involved pathways and potential bottlenecks within them can also be identified using omics approaches. In *A. nidulans*, transcriptome analysis combined with flux and physiology data was used to propose a model describing the competition between biomass formation and polyketide production for the available acetyl coenzyme A (Panagiotou et al. 2009).

### Comparison of production and wild-type strains

The extensive random mutagenesis in strain improvement programs generated production strains that differ substantially from their ancestral wild type at the genomic level. Genome rearrangements were previously demonstrated by pulsed-field gel electrophoresis (Walz and Kück 1991). The current availability of genome sequences from wild-type and production strains of a broad range of industrial filamentous fungi identified genome differences down to nucleotide resolution. Identified changes can be valuable indicators for targeted strain improvement using molecular genetic tools.

Comparison of the genome sequences from the *T. reesei* production strains NG14 and RUT-C30 with the wild-type strain QM6a revealed 223 SNPs, 15 small deletions or insertions, and 18 larger deletions responsible for the loss of more than 100 kb (Le Crom et al. 2009). More than half of the single nucleotide polymorphisms (SNPs) introduced within coding sequences are non-synonymous and can therefore lead to changes in protein functionality. A few of these changes were analyzed in more detail, for example, truncation of the *gls2α* gene encoding a β-glucosidase subunit in the production strain RUT-C30 (Geysens et al. 2005). Furthermore, the truncation of the transcription factor Cre1, caused by a premature stop codon introduced in the encoding gene leads to a loss of carbon catabolite repression (Ilmen et al. 1996). This enables the production of cellulases using glucose as a carbon source, which prevents cellulase production in wild-type strains. These findings are applicable to other industrially relevant fungi. It was, for instance, shown that deletion of the *cre1* gene can be used to increase penicillin titers in *P. chrysogenum* production strains (Cepeda-García et al. 2014). However, not every genomic alteration introduced by classical strain improvement using random mutagenesis can be connected to improved product formation. Production strains of *T. reesei* have lost a genomic fragment with a size of 85 kb compromising 29 genes (Seidl et al. 2008). But the loss of these fragments has no effect on cellulase production (Vitikainen et al. 2010). An alternative approach is the comparison of strains that are unable to synthesize a given product. The genome sequence of a cellulase-negative *T. reesei* strain revealed an SNP in the *xyr1* gene leading to a truncated and biologically inactive transcription factor (Lichius et al. 2015).

In *P. chrysogenum*, comparative genomics was used to analyze strain improvement processes. In a comparison of the genomes of the wild-type strain NRRL1951 with two derived production strains, a total of 558 SNPs were identified in coding regions leading to changes in the amino acid sequences of the encoded proteins (Salo et al. 2015). In this comparison, the authors identified SNPs in 29% of the secondary metabolite cluster. Besides the deactivation of secondary metabolite clusters, no enrichment of functional categories of mutated genes in production strains was observed. This led to the hypothesis that these clusters were inactivated in favor of β-lactam biosynthesis. An example of such inactivation is the sorbicillinoid biosynthesis cluster, which is involved in the biosynthesis of a yellow-colored pigment produced in wild-type strains. The formation of this class of pigments can hamper the downstream purification of secondary metabolites and therefore was also eliminated in other industrial filamentous fungi such as *A. chrysogenum* and *T. reesei* (Derntl et al. 2016).

The genetic alterations in production strains in comparison to the wild type were a relevant aspect of strain improvement programs for a long time. So far, SNP analysis has not provided substantial evidence for the preferred mutagenesis of genes from distinct functional categories.

An alternative approach to decipher genetic alterations in production strains was recently successfully conducted. The comparative transcriptional analysis of the wild-type and production strains from the two antibiotic producers *A. chrysogenum* and *P. chrysogenum* (Terfehr et al. 2017), revealed that both only distantly related industrial fungi show common transcriptional adaptions towards high-level β-lactam antibiotic biosynthesis. For example, industrial strains from both fungi show an enrichment of differentially regulated genes associated with the supply of precursors and energy for β-lactam production. Furthermore, genes required for pathways and cellular functions that are not needed for antibiotic production are downregulated. In addition, the effect of Velvet, a major regulator of secondary metabolism in filamentous fungi, was investigated in production strains of *A. chrysogenum* and *P. chrysogenum*. This global regulator controls approximately 50% of all secondary metabolite clusters. Most importantly, strain improvement and Velvet affect the expression of a large set of genes in a similar manner in both improved industrial fungi. Comparisons of production and wild-type strains can be also conducted at the protein level. Using two-dimensional electrophoresis, it was possible to identify protein level changes between a wild-type strain and two production strains with moderate and high penicillin biosynthesis capacities (Jami et al. 2010). The two production strains have undergone a global metabolic reorganization. They showed an overrepresentation of enzymes involved in penicillin precursor and energy metabolism, while proteins involved in stress response, virulence, and the biosynthesis of other secondary metabolites were nearly lost. A similar comparison with *A. chrysogenum* strains also identified comparable changes (Liu et al. 2015). Since thiamine biosynthesis enzymes are overrepresented in the production strain, this metabolism seems to be beneficial for cephalosporin C biosynthesis in this fungus. These conclusions are based on knock-down and overexpression studies and provide the potential to identify targets for strain improvement.

Besides understanding differences in secondary metabolism, proteome studies are used to identify bottlenecks during the secretion of recombinant proteins. The comparison of *A. nidulans* strains, showing different capacities of recombinant protein production, disclosed alterations in proteins related to amino acid metabolism, ribosome biogenesis, translation, and endoplasmic reticulum and provides targets for rational strain improvement (Zubieta et al. 2018). Strains used for the comparisons mentioned above can be derived from long-term strain improvement programs, but also from forward genetic approaches. Using the latter approach together with whole genome sequencing enables the identification of the low-affinity glucose transporter MstC from *A. niger*, whose deletion improved secretion of heterologous proteins (Reilly et al. 2018).

A different approach was used for *Aspergillus* species. Here, a genus-wide comparative genomics approach was used to identify genomic differences concerning primary and secondary metabolism, stress response, biomass degradation, and signal transduction (de Vries et al. 2017). In the case of organic acid production, the authors identified a link between the number of isoenzymes and the phylogeny of the analyzed fungi. Members from the section *Nigri* possess extra isoenzymes relevant for citrate and gluconic acid production, which correlates with the increased capacity of these strains to produce organic acid.

### Optimizing endogenous metabolic pathways

The identification of competing metabolic pathways can be a valuable approach for strain improvement by genetic manipulation. In this context, pathways that stay in direct competition because of using the same substrate and pathways that degrade the desired product are of great interest. In addition to competing pathways, transport-associated processes can also be a bottleneck. Comparisons of strains with different characteristics concerning these problems and basic research can provide hints for optimization.

Because of the high secretion capacity, filamentous fungi are broadly used in industry to produce endogenous and heterologous products, particularly, *A. oryzae*, *A. niger*, and *T. reesei*. However, these fungi also secrete a couple of proteases which will degrade the products of interest and thus may diminish the maximum product yield. Therefore, protease-deficient *Trichoderma* and *Aspergillus* strains were generated to circumvent this problem. Using *A. oryzae* strains that lack two proteases, the heterologous production of human lysozyme and bovine chymosin was further improved by deletion of additional protease-encoding genes. A consequence was the generation of a protease-deficient strain with an enhanced capability of heterologous protein production (Yoon et al. 2011). In a similar approach with *T. reesei* strains, the deletion of nine protease-encoding genes led to a doubled interferon alpha-2b production (Landowski et al. 2016). These yields are comparable to those achieved with *E. coli*, where the product must be refolded from inclusion bodies. The *T. reesei* system even outperforms the *Pichia pastoris* system, which was used frequently for interferon production. Another option to decrease extracellular protease levels is the modification of the underlying gene regulation. The transcription factor PrtT from *A. niger* is applicable for this approach since it regulates the expression of extracellular protease-encoding genes (Punt et al. 2008). The abovementioned strategies, developed for well-known production hosts, may be transferred to less-established production organisms. Genome sequencing of the thermophilic filamentous fungus *Myceliophthora thermophila* and subsequent identification of protease-encoding genes were used to generate protease-deficient strains for cellulase production (Berka et al. 2011; Szabo et al. 2013).

Misfolding is another problem in the production of heterologous proteins. Based on transcriptomic data, it was shown that the biosynthesis of heterologous proteins activates the unfolded protein response (UPR) in *A. niger* (Guillemette et al. 2007; Kwon et al. 2012). The activation of UPR leads to proteolysis, which can reduce the product yield substantially. Using the transcriptomic data, it was possible to identify core genes involved in this pathway as promising targets for strain improvement. The amount of secreted heterologous proteins was increased by deletion of the *doaA* gene, encoding an essential factor for ubiquitin-mediated proteolysis. This was further optimized by overexpression of the *sttC* gene, encoding a protein involved in the glycosylation of secreted products (Jacobs et al. 2009).

Autophagy is a highly conserved intracellular pathway that degrades non-functional or not needed cellular components. This process may affect high yield expression of proteins and is thus a promising target for strain improvement programs. In chymosin-producing *A. oryzae* strains, the inactivation of autophagy leads to a threefold increase in product formation (Yoon et al. 2013). Besides heterologous protein production, secondary metabolite production can also be affected by autophagy. Autophagy-deficient *P. chrysogenum* strains have an increased amount of penicillin biosynthesis enzymes and thus synthesize more penicillin (Bartoszewska et al. 2011). Similarly, the lack of Atg1, which is essential for autophagosome formation, led to increased cephalosporin C production rates in *A. chrysogenum* strains (Wang et al. 2014). However, the modification of autophagy does not necessarily lead to increased product yields. The deletion of *Acatg11* results in autophagy deficiency and a reduced cephalosporin C titer in the corresponding *A. chrysogenum* strain (Liu et al. 2017a).

Protein degradation can also be a problem if the desired heterologous product is usually catabolized by the host organism. In order to produce galactaric acid using *A. niger*, a gene coding for a uronate dehydrogenase of prokaryotic origin was introduced into the genome. In addition to that, endogenous pathways for galactaric acid degradation were identified using RNA-seq data and inactivated using the CRISPR/Cas9 system (Kuivanen et al. 2016).

### Product modification for optimized application

Products synthesized by filamentous fungi are used in a broad area of applications. This area ranges from proteases and lipases in washing agents in private households to the industrial scale use of enzymes, e.g., cellulases for bioethanol production. Based on the diverse areas of application, the products must meet different criteria for different physical and chemical parameters. While enzymes in washing agents often need ambient temperature optima, the vast majority of industrial used enzymes should be thermotolerant (Struvay and Feller 2012). High temperatures offer advantages, e.g., faster conversation rates, a reduced risk of contamination, and an increased mass transfer (Grigoriev et al. 2011). However, even heat lability can also be beneficial because these enzymes can be easily inactivated by a moderate increase in temperature. Another crucial parameter for the activity and stability of enzymes is the pH optimum. For instance, acid-stable cellulases are highly required for bioethanol production, because substrates are often treated with acids prior to the enzymatic processes. Functional analysis of genomes and even metagenomes from extremophiles can help to identify enzymes that meet these criteria, even if the original host cannot be used for production.

Phytases, phosphohydrolases that catalyze the hydrolysis of phytic acid, releasing bioavailable phosphorus, were optimized based on various homologous phytase-encoding genes. The construction of a consensus phytase gene led to an enzyme with improved activity and thermostability (Lehmann et al. 2000; Tomschy et al. 2000). In *T. reesei*, the temperature optimum of endoglucanase was increased by 16 °C in a similar approach (Trudeau et al. 2014). Besides optimizing enzymes to fit certain criteria, one can also directly choose enzymes from extremophiles. The comparative genomic analysis of the two thermophilic biomass-degrading fungi *Myceliophthora thermophila* and *Thielavia terrestris* disclosed a wealth of thermostable enzymes for hydrolysis of all major polysaccharides occurring in biomass (Berka et al. 2011). Via the heterologous expression of a thermostable β-glucosidase of *Neosartorya fischeri*, a closely related species to *A. fumigatus*, the cellulytic activity of *T. reesei* was improved (Xue et al. 2016).

Another alternative strategy is the use of metagenome libraries. Using a library derived from deep-sea sediments, a cold-active lipase was identified with an optimal activity at 25 °C (Zhiwei et al. 2015). Furthermore, a metagenomic fosmid library obtained from the microbial community of a biogas digester provided a new cellulase gene, which was fused with the *cbh1* gene from *T. reesei*. Rut-C30 strains expressing this enzyme showed a higher cellulytic activity and concentrations of hydrolytically released glucose increased by about 20% (Geng et al. 2012). Besides the search for enzymes with higher activity, metagenomics can also be used to identify enzymes with new substrate specificities.

## Conclusion

Over four decades ago, the DNA-mediated transformation systems for filamentous fungi were first described. Since then, many different molecular tools were developed that provide the necessary basis to manipulate filamentous fungi for a broad range of biotechnical applications. However, we are still searching for promoter systems that have strong and tuneable expression properties, not only in a few host systems but rather in a broad range of biotechnically used fungi. Another central question concerns the functionality of heterologous proteins. For example, the post-translational protein modification is the main problem when human protein genes are functionally expressed in diverse fungal hosts. We still await tightly regulated expression systems that allow the controlled glycosylation of mammalian proteins. We foresee that the growing information obtained from functional genomics regulatory annotation data combined with transcriptional expression assays can identify complex regulatory networks that allow the synthesis of secondary metabolites, which still remain to be discovered.
